# Comparing K-mer based methods for improved classification of 16S sequences

**DOI:** 10.1186/s12859-015-0647-4

**Published:** 2015-07-01

**Authors:** Hilde Vinje, Kristian Hovde Liland, Trygve Almøy, Lars Snipen

**Affiliations:** 1 Department of Chemistry, Biotechnology and Food Sciences, Norwegian University of Life Sciences, Oslo, N-1432 Ås Norway; 2 Nofima AS, Osloveien 1, Oslo, 1430 Ås Norway

## Abstract

**Background:**

The need for precise and stable taxonomic classification is highly relevant in modern microbiology. Parallel to the explosion in the amount of sequence data accessible, there has also been a shift in focus for classification methods. Previously, alignment-based methods were the most applicable tools. Now, methods based on counting *K*-mers by sliding windows are the most interesting classification approach with respect to both speed and accuracy. Here, we present a systematic comparison on five different K-mer based classification methods for the 16S rRNA gene. The methods differ from each other both in data usage and modelling strategies. We have based our study on the commonly known and well-used naïve Bayes classifier from the RDP project, and four other methods were implemented and tested on two different data sets, on full-length sequences as well as fragments of typical read-length.

**Results:**

The difference in classification error obtained by the methods seemed to be small, but they were stable and for both data sets tested. The Preprocessed nearest-neighbour (PLSNN) method performed best for full-length 16S rRNA sequences, significantly better than the naïve Bayes RDP method. On fragmented sequences the naïve Bayes Multinomial method performed best, significantly better than all other methods. For both data sets explored, and on both full-length and fragmented sequences, all the five methods reached an error-plateau.

**Conclusions:**

We conclude that no *K*-mer based method is universally best for classifying both full-length sequences and fragments (reads). All methods approach an error plateau indicating improved training data is needed to improve classification from here. Classification errors occur most frequent for genera with few sequences present. For improving the taxonomy and testing new classification methods, the need for a better and more universal and robust training data set is crucial.

## Background

The exploration of microbial communities is now a major focus in microbiology, opening new approaches to the study of microbiomes of humans and other organisms as well as the communities found in natural environments of air, water or soil [1]. Already in the 1980s Carl Woese introduced the rRNA-based phylogenetic comparisons of prokaryotes [2, 3], and the 16S rRNA gene is still the most useful genomic marker for the study of diversity and composition of metagenomes. The classification of 16S sequences obtained from some samples is a classical pattern recognition problem, i.e. recognizing some pattern in a sequence and assign it to one out of several predetermined categories. Whether the sequences are subjected to multiple alignments or, as in this paper, counting of short words, some assignment must be made based on how similar these sequences are to previously classified sequences. Naturally, the methods employed should give as accurate classifications as possible, but in metagenomics time-efficiency is also an issue since the number of sequences to classify may be vast. It should also be noted that with today’s massively parallel sequencing technologies, shorter reads covering only a region of the gene are more accessible [4–6], making classification methods that perform well on sequence fragments essential.

However, classifications based on 16S rRNA sequences do not only have a practical use in metagenomics. In fact, this marker is used to build the entire prokaryotic taxonomy and is considered the gold standard for phylogenetic studies [7–9]. In this perspective the classification of full-length 16S sequences is the issue. It should also be noted that in this context we should make all possible efforts to have the absolute best classifications available, and time-efficiency is no longer important.

A number of different procedures have been used to classify 16S sequences, and several different databases purposely designed as 16S rRNA repositories are available, e.g. Greengenes [10], RDP [11] and SILVA [12]. Most procedures for taxonomic studies have been based on alignments and reconstruction of phylogenetic trees, making use of some predefined evolutionary models and relevant algorithms [3, 13, 14]. However, with the enormous increase in data from next generation sequencing technology, these approaches suffer some problems. First, the computational time required to align a large set of sequences increases exponentially by its size. Secondly, greedy algorithms of some kind are required to construct these huge alignments and these sparse, monolithic alignments will most likely contain a substantial number of errors due to the heuristics employed. Finally, the lack of consensus, e.g. on evolutionary model assumptions, has made it impossible to arrive at an official taxonomy for prokaryotes, the most widely accepted taxonomy being the Bergey’s Manual of Systematics of Archaea and Bacteria [15]. Thus, objective pattern recognition algorithms are likely to be valuable tools for building the prokaryotic taxonomy itself.

The most popular pattern recognition methods for 16S sequences are those based on counting *K*-mers, i.e. overlapping ‘words’ of length *K* in the sequences [16–19]. Wang et al. [19] developed the RDP classifier, based on the naïve Bayes principle and a word-length of *K*=8. The RDP classifier is now close to being a standard in 16S based classification, and was in 2011 selected by Essential Science Indicators as the most-cited paper in a highlighted research area of microbiology [20]. *K*-mer methods are fast and will not suffer from the same uncertainties as the procedures based on evolutionary models and alignments. This way of converting sequences to numerical data is not as intuitive as evolutionary models, and lack the obvious interpretation given by evolutionary distances, but they are very objective in their mechanism. Also, in a previous study [21] we found that in order to obtain the best possible classification at the genus level, one has to consider more or less all positions along the full-length 16S sequences (around 1500 bases), not only hyper-variable regions or other subsequences. This is another advantage of the *K*-mer methods; they use all data in asequence.

However, *K*-mer based pattern recognition methods are not without model assumptions, and the RDP classifier uses the *K*-mer counts in one out of a number of alternative ways. Recent suggested improvements of this approach [22] have made it necessary to make a more systematic investigation on how well other *K*-mer based methods would perform, and possibly to reveal how and where efforts should be made to improve the objective classification of prokaryotes. In this paper we have compared different classification methods based on *K*-mer data for 16S sequences. We consider five different methods based on different machine-learning approaches, and we have compared their performance for full-length sequences as well as fragments. In addition to the method comparison, we also try to pinpoint where improvements should be made in order to give us better future methods for the important problem of identifying the majority of species on thisplanet.

## Methods

### Data

To compare methods we used two data sets. The Trainingset9 is the data used to compare 16S classification methods in [19], and was downloaded from RDP [11]. It consists of 10032 16S rRNA sequences varying from 320 to 2210 bases in length, with the majority around 1400 bases. There are 37 phyla and 1943 genera represented in this set.

The SilvaSet is an extract from the SILVA database [12], where the largest genera have been ‘pruned’ by random sampling to contain fewer sequences. This set has 29520 sequences, covering 29 phyla and 1533 genera. The main reason for including this data set is that it is a manually curated data set different from Trainingset9, which was used during the development of the RDP-classifier.

In this paper we only consider classification to genus, i.e. the lowest taxonomic level of these data. This is the most challenging and also the most relevant problem for most studies where taxonomic classification is important.

**Fig. 1:**
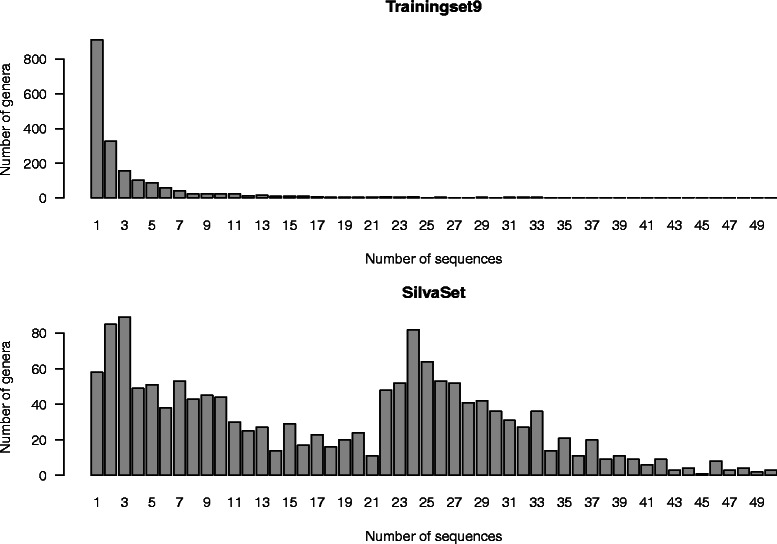
The size distibution histogram of genera in Trainingset9 and SilvaSet. The panels show the size distibution of genera in Trainingset9 and SilvaSet. Genera with more than 50 sequences are not included here, but in Trainingset9 there are 26 and in SilvaSet there are 40 such genera

The distributions of sequence abundance across genera are skewed for both Trainingset9 and SilvaSet. Genera with only one sequence available are by far the most common in Trainingset9 (Fig. 1). These singleton genera were included in the analysis, but will always be mis-classified by all methods, and all reported errors exclude these sequences. For Trainingset9 few genera have more than 15 sequences, while some genera are considerably larger (not shown). The genus with most sequences is *Streptomyces*, which consists of 513 sequences. In the SilvaSet the difference in genus sizes is not as pronounced as in Trainingset9, but the majority of genera consists of 40 or less sequences. The genus with most sequences is *Pseudomonas* with 115 sequences.

To estimate the model performance we conducted a 10-fold cross validation [23] for all methods. The data were ordered alphabetically by genus name and split into ten different segments by enumeration from one to ten repeatedly, and then assigned to segments according to this number, i.e. every tenth sequence belongs to the same segment. This ensured a maximum spread of all genera across the segments. Each segment was set aside once as a test set, while the rest were used as training set in each cross-validation iteration.

### K-mer based methods

All methods compared here represent a 16S sequence by its overlapping *K*-mers, i.e. words of length *K*. There are *D*=4^*K*^ possible words of length *K* in the DNA (RNA) alphabet, and in our study we tested word lengths from two to eight. The methods tested differ in the way they represent a sequence as K-mers and how this information is utilized in a statistical learning algorithm to achieve best possible classification.

All five methods were implemented in the software environment R [24]. Our implementation of the RDP classifier was tested against the original Java-implementation to ensure consistency. The PLS and nearest-neighbour methods already exist in the R-environment.

### RDP

The RDP method considers only the presence/absence of a word in a sequence, not its frequency. All words of length *K* are ordered alphabetically as *w*_1_,*w*_2_,…,*w*_*D*_. For every sequence, we create a vector of *D* elements where element *j* is 1 if word *w*_*j*_ is present in the sequence, and 0 if not. We have chosen to describe the RDP method in detail below, even if this has been done in [19], because this method serves as a reference for the other methods described later.

#### Training

For each of the *N* sequences in the training set we get a vector of 1’s and 0’s, and these vectors are arranged as rows in the *N*×*D* matrix ***A*** ^*rdp*^.

First, we estimate the unconditional probability: The probability of presence of each word regardless of genus. Summing the elements in each column of ***A*** ^*rdp*^ produces the vector *n*_1_,*n*_2_,…,*n*_*D*_, i.e. *n*_*j*_ is the number of sequences in the training set where word *w*_*j*_ is observed at least once. The probability that word *w*_*j*_ will be found present in any sequence is estimated by

(1) $$ Pr(w_{j}) = \frac{n_{j} + 0.5}{N+1}   $$

where the added 0.5 and 1 guarantees that no probability is zero or one.

Next, consider only sequences from genus *g*, i.e. we consider a sub-matrix $\textit {\textbf {A}}^{rdp}_{g}$ containing only the *M*_*g*_ rows corresponding to genus *g*. Again we can sum over the rows of $\textit {\textbf {A}}^{rdp}_{g}$, and we get the vector *m*_*g*,1_,*m*_*g*,2_,…,*m*_*g*,*D*_, i.e. *m*_*g*,*j*_ is the number of sequences from genus *g* where we observe the word *w*_*j*_ at least once. The genus-specific or conditional probabilities are estimated by

(2) $$ q_{g,j} = Pr(w_{j}|g) = \frac{m_{g,j}+Pr(w_{j})}{M_{g}+1}   $$

If the training set contains data for *G* genera, we can arrange the probabilities *q*_*g*,*j*_ in a *G*×*D* matrix ***Q*** ^*rdp*^ where the element in row *g* and column *j* is *q*_*g*,*j*_, for *g*=1,…,*G*, *j*=1,…,*D*. This matrix ***Q*** ^*rdp*^ is the trained model, with a set of probabilities (a row) for each genus.

#### Classification

Given a new sequence we construct the vector ***a*** corresponding to a row in the matrix ***A*** ^*rdp*^ from above. Element *j* in ***a*** is 1 if word *w*_*j*_ is found in the new sequence, and 0 otherwise. The unconditional probability of ***a*** is found from (1) by

(3) $$ Pr(\textit{\textbf{a}}) = \prod_{j=1}^{D} Pr(w_{j})^{a_{j}}   $$

where *a*_*j*_ is element *j* in ***a*** and *p*_*j*_ is from (1). Notice that *P* *r*(***a*** is a joint probability of observing the words we see in this sequence. The naïve Bayes approach lies in the assumption that this joint probability can be written as a product of the marginal probabilities, as we have done on the right hand side above. This assumption is correct only if the elements of ***a*** are independent, which is a naïve assumption, but often still works in a satisfactory manner.

The conditional probability of ***a*** given some genus *g* is computed in a similar way from (2) by

(4) $$ Pr(\textit{\textbf{a}}|g) = \prod_{j=1}^{D} q_{g,j}^{a_{j}}   $$

From the general relation between conditional and marginal probabilities it follows that

(5) $$ Pr(g|\textit{\textbf{a}}) = \frac{Pr(g)Pr(\textit{\textbf{a}}|g)}{Pr(\textit{\textbf{a}})}   $$

where the probability on the left hand side is the criterion we use to classify. This is the *posterior probability* of genus *g* given the observed sequence ***a***, and we classify to the genus that maximizes this probability. On the right hand side we have the *prior probability* of genus *g*, *P* *r*(*g*), in addition to the two probabilities we computed in (3) and (4). It is customary to set the prior probability equal to the proportion of data from genus *g* in the training data set. In the RDP classifier the prior probabilities are assumed to be equal for all genera, and genera with few sequences are just as likely to be observed as those with many sequences in the training set. In our study we considered both flat priors (RDP) as well as priors proportional to genus abundances.

The posterior probability *P* *r*(*g*|***a***) is computed for every genus, and we assign the sequence to the genus where we get the largest probability. Notice that the denominator *P* *r*(***a***) in (5) does not depend on genus *g*. Hence, the *g* that maximizes *P* *r*(*g*|***a***) is exactly the same *g* that maximizes *P* *r*(***a***|*g*)*P* *r*(*g*), and we can ignore *P* *r*(***a***) altogether. Also, if the prior probabilities *P* *r*(*g*) are identical for all genera, we get the simple relation *P* *r*(*g*|***a***)=*P* *r*(***a***|*g*).

From a computational perspective, we prefer the log-transformed version of (5) (ignoring *P* *r*(***a***)), and using the relation in (4) we get

(6) $$ \log_{2}(Pr(g|\textit{\textbf{a}})) = \log_{2}(Pr(g)) + \sum_{j=1}^{D} a_{j}\log_{2}(q_{g,j})   $$

since this log-probability is maximized for the same *g* as the one in (4). If the matrix ***Q*** ^*rdp*^ from the training step is log-transformed and called ***L*** ^*rdp*^, and ***p*** is the column-vector of the *G* log-priors for all genera, we can compute the *score* vector

(7) $$ \textit{\textbf{z}} = \textit{\textbf{p}} + \textit{\textbf{L}}^{rdp} \cdot \textit{\textbf{a}}^{\prime}   $$

as the inner product of ***L*** ^*rdp*^ and the column vector ***a*** ^′^. The score vector ***z*** has one element for each genus, and we assign to the genus where ***z*** has its maximum value. In case of two or more genera obtaining the same maximum value, the sequence is marked as unclassified.

Notice that with flat priors, the terms log2(*P* *r*(*g*)) are identical for all *g*, i.e. all elements of ***p*** are identical, and it can be omitted from (7) since it will add the same to all genera.

### Multinomial

The Multinomial method differs from the RDP method by considering the relative frequency of every word instead of presence/absence. The naïve Bayes principle is the same. A similar approach has also been tested by Lui and Wong in their work in [22].

#### Training

For each of the *N* sequences in the training set we get a vector of frequencies, i.e. element *j* is the number of times we observe *w*_*j*_ in the sequence. These vectors are arranged as rows in the *N*×*D* matrix ***A*** ^*frq*^.

As before we consider a sub-matrix $\textit {\textbf {A}}^{frq}_{g}$ containing the *M*_*g*_ rows corresponding to genus *g*. Summing over the columns of $\textit {\textbf {A}}^{frq}_{g}$ we get a vector *m*_*g*,1_,*m*_*g*,2_,…,*m*_*g*,*D*_. The genus-specific frequencies *F*(*w*_*j*_|*g*) are:

(8) $$ F(w_{j}|g) = \frac{m_{g,j}}{M_{g}} + \frac{1}{D}   $$

where $\frac {1}{D}$ pseudo-counts are added to each frequency to avoid 0 counts. The multinomial probabilities for genus *g* is then calculated by dividing each *F*(*w*_*j*_|*g*) by their respective row sum, giving us a new set of multinomial probabilities *q*_*g*,*i*_:

(9) $$ q_{g,j} = F(w_{j}|g)/\sum_{k=1}^{D}F(w_{k}|g)   $$

The trained model consists of the (*G*×*D*) matrix ***Q*** ^*mlt*^ where row *g* contains the multinomial probabilities *q*_*g*,*j*_ for genus *g*.

#### Classification

From the new sequence we construct the frequency vector ***a*** corresponding to a row in the matrix ***A*** ^*frq*^ above. Again we use the naïve Bayes approach to compute a scorevector ***z***:

(10) $$ \textit{\textbf{z}} = \textit{\textbf{p}} + \textit{\textbf{L}}^{mlt} \cdot \textit{\textbf{a}}^{\prime}  $$

where ***L*** ^*mlt*^ is the log-transformation of ***Q*** ^*mlt*^ from the training step and ***p*** are the log-priors just as for the RDP-classifier. The score vector ***z*** has one element for each genus, and the sequence is assigned to the genus with maximum score in ***z***. In case of two or more genera obtaining the same maximum value, the sequence is marked as unclassified.

### Markov

In the present context ordinary Markov models consider word frequencies, but differ from the naïve Bayes principle used by the previous two methods. Markov models have been tested on sequence data with the *K*-mer approach in earlier studies, e.g. by Davidsen et al. [25].

#### Training

The training step corresponds to estimating the transition probabilities of the Markov model. Any word of length *K* can be split into the *pretext* consisting of the first *K*−1 symbols, and the last letter, being A, C, G or T. The transition probabilities are the conditional probabilities of the last letter given the pretext. These probabilities are usually organized in a transition matrix with 4 columns (one for each letter) and one row for each pretext (4^*K*−1^ rows). However, these probabilities can equally well be organised in a single row-vector, where the conditional probabilities of A given the ordered pretexts is found at positions *I*_*A*_=(1,5,9,…), for C given the ordered pretexts in positions *I*_*C*_=(2,6,10,…) and so on. Note that this corresponds to the *K*-mers in alphabetical order. Each consecutive four positions corresponds to the same pretext, extended by A, C, G and T, respectively.

The matrices ***A*** ^*frq*^ and $\textit {\textbf {A}}^{frq}_{g}$ are computed as for the Multinomial method. Summing over the columns of $\textit {\textbf {A}}^{frq}_{g}$ again produces genus-specific frequencies *F*(*w*_*j*_|*g*) as in (8). If *K*-mer *w*_*j*_ contains pretext *h* followed by, say, A, then the corresponding genus-specific transition probability is estimated by

(11) $$ q_{g,j} = F(w_{j}|g)/\sum_{k\in I_{A}}F(w_{k}|g)  $$

and similar if the pretext is followed by C, G or T, *I*_*A*_ is replaced by the corresponding index set. If we had organized the transition probabilities in a matrix, this value would appear in cell (*h*,1) since we consider pretext *h* followed by A (column 1). Instead we arrange these probabilities in a row vector of *D* elements. Having the transition probabilities for each genus, we arrange the vectors as rows in a (*G*×*D*) matrix ***Q*** ^*mrk*^. The latter organization of the transition probabilities is done only to have the same data structure as for the other methods; it does not affect the computations.

#### Classification

From the new sequence we count *K*-mers as for the Multinomial method, constructing the frequency vector ***a*** corresponding to a row in the matrix ***A*** ^*frq*^. We compute scores for the sequence as

(12) $$ \textit{\textbf{z}} = \textit{\textbf{L}}^{mrk} \cdot \textit{\textbf{a}}^{\prime}  $$

where ***L*** ^*mrk*^ is the log-transformation of ***Q*** ^*mrk*^. Again we classify to the genus yielding maximum score. In case of a tie, the sequence is marked as unclassified.

### Nearest-neighbour (NN)

In this method we use nearest-neighbour classification based on multinomial probabilities. Nearest-neighbour methods have no specific training step, but use the training data as a database and perform a lookup based on some characteristics of the query sequence. Another 16S nearest-neighbour method, called the Similarity Rank tool, was published by Maidak et al. [26] for use in The Ribosomal Database Project.

As before we compute the (*N*×*D*) matrix ***A*** ^*frq*^ by word counting, where *N* is the number of sequences in the training set. Then we divide all elements in a row by its row-sum to obtain multinomial probabilities, and these are stored in the (*N*×*D*) matrix ***A*** ^*mlt*^. Thus, each training sequence, with its labelled genus, is represented as a row in this matrix.

For every new sequence we also count word frequencies and divide by the number of words in the sequence, producing a vector ***a*** similar to a row in ***A*** ^*mlt*^. The Euclidean distance from ***a*** to all sequences (rows) in the training set is computed. The new sequence is assigned to the same genus as the nearest neighbour in the training set. In case of a tie, i.e. two or more genera are nearest neighbours, it is left unclassified.

### Preprocessed nearest-neighbour (PLSNN)

In this method we extend the nearest-neighbour by combining it with the partial least squares (PLS) method [27]. This is a supervised learning method that has been used in many bioinformatics applications (e.g. [28–32]). A reason for the wide-spread use of PLS is that it is especially applicable when we have many correlated explanatory variables, which is typical for the present *K*-mer data, especially as *K* increases.

The idea is to compute a linear mapping from the *K*-mer frequency space to a much lower dimensional space, and then look for nearest-neighbours in this low-dimensional space. In *K*-mer space every sequence has *D*=4^*K*^ coordinates, and in the nearest-neighbour method above all coordinates (*K*-mers) have equal weight. However, it is more than likely that some of these will be more or less important for recognizing a particular genus. Replacing the original *D* dimensional space by a smaller number of combinations can be seen as a preprocessing of the data before the nearest-neighbour step, hopefully resulting in more ‘correct’ distances between sequences when seeking the nearest neighbour.

#### Training

From the training data we again compute the (*N*×*D*) matrix ***A*** ^*mlt*^ as above. This is used as the matrix of explanatory variables in training the PLS-method. The response is the genus for each sequence. This is coded as a row-vector of *G* elements, with 1 in position *g* if the sequence comes from genus *g* and 0 in all other positions. This assembles into an (*N*×*G*) matrix ***Y***.

The PLS assumption is based on the linear model

(13) $$ E(\textit{\textbf{Y}}) = \textit{\textbf{A}}^{mlt} \boldsymbol{\beta}   $$

where ***β*** is some (*D*×*G*) vector of regression coefficients. The algorithm will search for an orthogonal sub-space by combining the variables (columns) of ***A*** ^*mlt*^ and maximising the covariance between ***Y*** and ***A*** ^*mlt*^. The algorithm first finds the 1-dimensional sub-space, then the 2-dimensional, etc. The main idea is to stop the search after *C* dimensions, where *C*<<*D* but still enough to have a good fit according to the model in (13). This means we end with

(14) $$ \textit{\textbf{A}}^{mlt} \approx \textit{\textbf{S}} \textit{\textbf{R}}^{\prime}   $$

where the (*N*×*C*) dimensional matrix ***S*** consist of linear combinations of the columns in ***A*** ^*mlt*^, and ***R*** is some orthonormal projection matrix. The rows of ***S*** are the training sequences represented in the *C*-dimensional subspace with maximum covariance to genus information. In this representation we have filtered out less important variation in *K*-mer frequencies, e.g. variation within genera. Distances between sequences in this space should be more sensitive to between-genus variation and less sensitive to within-genus variation. For every word length *K* we tested 8 different dimensions *C*. The maximum was set to *C*_*max*_= min(*N*−1,*D*−1,2000), and we used *C*=*i* *C*_*max*_/8 for *i*=1,2…,8.

#### Classification

For every new sequence we compute a vector ***a*** similar to a row in ***A*** ^*mlt*^. From (14) it follows that ***A*** *m* *l* *t* ***R***≈***S*** since ***R*** is orthonormal, and thus we can compute ***s***=***a*** ***R***. The vector ***s*** is the representation of the new sequence in the subspace spanned by ***S***. The new sequence is finally classified with the nearest-neighbour method as before, where Euclidean distances from ***s*** to all rows of ***S*** areconsidered.

## Results and discussion

We have tested five methods for *K*-mer based classification of 16S sequences, using a 10-fold cross validation, on two different data sets to compare their performance.

**Fig. 2:**
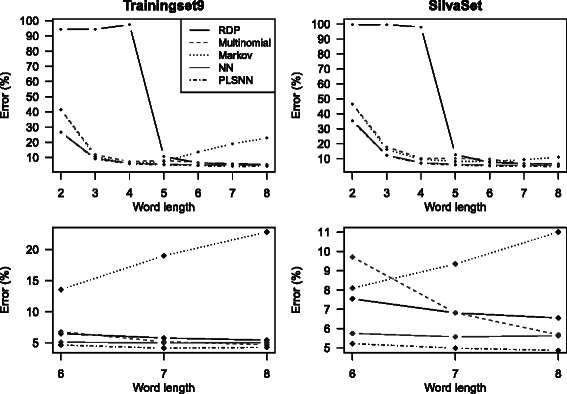
Classification error for full-length 16S sequences. The top panels display the classification error for full-length sequences using all methods on word lengths *K*2−*K*8. The bottom panels are the same results only zoomed at the last three word lengths (*K*6−*K*8). Hence, the results are discrete values for every *K*-mer length and the connecting lines are merely to aid visual interpretation

Figure 2 shows the classification error for full-length sequences for both Trainingset9 and the SilvaSet. The Multinomial, the NN and the PLSNN method, all had a smooth, steady reduction in classification error from word length three, while the RDP method did not stabilize until word length five. The latter is due to the present/absent logic of this method: With too short word-length almost all words are present in most sequences. RDP and Multinomial had their minimum error at word length eight, NN and PLSNN at word length seven for Trainingset9 and eight for the SilvaSet, while The Markov method reached the minimum error rate at word lengths of four and six, respectively, for the two data sets. The smallest error reached is fairly similar for all methods. The minimum error level was around 5 *%* for Trainingset9, and slightly higher for the SilvaSet.

**Fig. 3:**
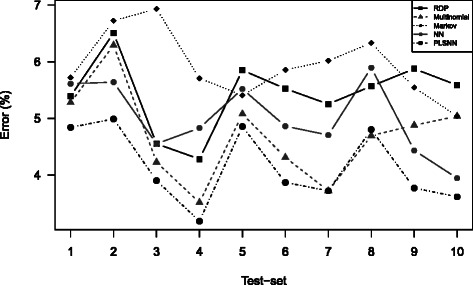
Classification error for each test-set. For Trainingset9 classification error for all the five methods are displayed for each of the 10 different test-sets from the 10-fold cross validation for full-length sequences. The SilvaSet gave similar results. Hence, the results are discrete values for every *K*-mer length and the connecting lines are merely to aid visual interpretation

The classification errors for the optimal word lengths are summarized in Table 1. For full-length sequences, using the optimal word length for each method, PLSNN performed best on both data sets with classification errors 4.2 *%* and 4.9 *%* respectively. The differences from the other methods may seem small, but were stable. This is indicated by the error percentages in each of the ten cross-validation test-sets (Fig. 3). Each test set was a random subset of the full data set. The fact that methods behave consistent across subsets is an indication of a stable difference. From Fig. 3 we observed that not only was the PLSNN method overall best, but also best in nine out of ten sub-sets. We also noticed that the RDP method was not among the best methods in any sub-sets, and the Markov method produced the largest error in most cases. To test the effect of methods on the classification error, we employed a standard analysis-of-variance, using method as fixed effect (five levels) and test set as random effect (ten levels). Using the RDP method as a reference method, we made a pairwise comparison with Tukey’s Honestly Significant test of the other four methods. The p-values are found in Table 2. The Markov method was significantly poorer and both the Multinomial and the PLSNN methods were significantly better (*p*<0.05) than RDP on full-length sequences for both data sets.

**Table 1:** Results from a 10-fold cross validation. Classification errors (% misclassified) for the different methods at their optimal word length and for various data sets. Singleton genera errors are not included since they add the same to all methods

	Trainingset9		SilvaSet	
Method	Full-length	Fragments	Full-length	Fragments
PLSNN	4.15 (K7)	16.96 (K8)	4.87 (K8)	24.33 (K7)
Multinomial	4.70 (K8)	16.00 (K8)	5.68 (K8)	19.73 (K8)
NN	4.99 (K7)	16.54 (K8)	5.63 (K8)	24.02 (K8)
RDP	5.43 (K8)	16.42 (K8)	6.55 (K8)	20.49 (K8)
Markov	5.93 (K4)	21.78 (K6)	8.10 (K6)	22.98 (K7)

**Table 2:** p-values for pairwise comparison of methods. Results from ANOVA on the effect of methods. The RDP is considered our control level and the p-values stated in the table below are the pairwise comparison for the four other methods to RDP

	Trainingset9		SilvaSet	
Method	Full-length	Fragments	Full-length	Fragments
PLSNN	<0.001(−)	0.002(+)	<0.001(−)	<0.001(+)
Multinomial	0.016(−)	0.026(−)	<0.001(−)	<0.001(−)
NN	0.293(−)	0.895(+)	<0.001(−)	<0.001(+)
Markov	0.198(+)	<0.001(+)	<0.001(+)	<0.001(+)

The signs in the parentheses indicate if a method gave smaller (−) or larger (+) errors than RDP

**Fig. 4:**
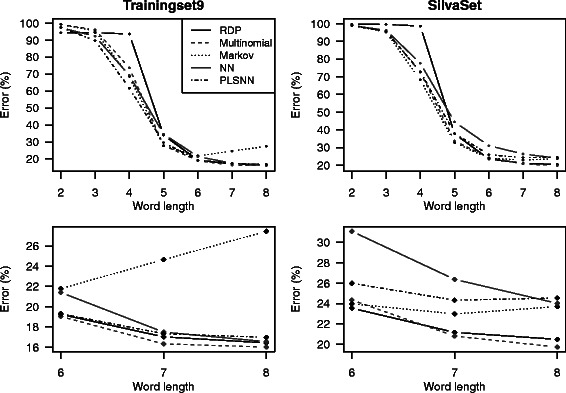
Classification error for fragments. The top panels display the classification error for sequence fragments using all methods on word lengths *K*2−*K*8. Sequences were split into 10 (partly overlapping) fragments of 200 bases, and all fragments were classified. The bottom panels are the same results only zoomed at the last three word lengths (*K*6−*K*8). Hence, the results are discrete values for every *K*-mer length and the connecting lines are merely to aid visual interpretation

All methods were also tested on shorter fragments of 16S sequences. Present sequencing technologies provide high-quality reads up to a few hundred bases, and some kind of assembly is required to provide a full-length 16S sequence (minimum 1200 bases). Thus, classification based directly from the reads is desired. We divided the test sequences into ten partially overlapping fragments of 200 bases, and all fragments were classified. Figure 4 shows the classification error based on the fragment sequences for both Trainingset9 and the SilvaSet. No method behaved well before word lengths of at least five or six, and again there was some error-plateau below which no method reached. Naturally, the errors were larger than for full sequences, since the information content of these shorter fragments must be smaller than the full sequence. Again, the ANOVA analysis was performed and we found that, compared to our control method RDP, the Multinomial was the only method that performed significantly better (*p*<0.05) for both data sets. PLSNN, on the other hand, now performed significantly poorer than RDP. The details of the results can be seen in Tables 1 and 2.

A difference between 4.2 *%* (full-length PLSNN Trainingset9) and 5.4 *%* (full-length RDP Trainingset9) error may seem small, but for building the taxonomy itself, there is no excuse for ignoring any improvement in methods. In principle the error should be zero. In a more practical use, where we want to classify a large number of sequences, a difference in 1 *%* means many misclassified sequences. Computation time is also an issue that should be taken into consideration. The RDP, Multinomial and Markov methods are fast and easy to both train and use for subsequent classification. All nearest-neighbour methods, including NN and PLSNN, are slower since they require distance computations for each new sequence to every sequence in the training set. The PLSNN method requires heavy computations during training, but once this has been done, new sequences are classified faster (and better) than with NN since distances are computed in a smallersub-space.

The Markov method appears to be the clear loser in our tests. Not only does it give poorest best-case results, but we also noticed that the best word length for the Markov classifier changed from four to seven depending on the data set. The uncertainty in word length makes this method unstable and unreliable and it is discarded as a fruitful approach for 16S sequence classification.

In the PLSNN method we employ the PLS method as a preprocessing of the count data, finding linear combinations of the *K*-mer counts having maximum class information. If we consider word length seven there are 4^7^=16384 different *K*-mers. A full-length 16S sequence has around 1500 words of this length, which means more than 90 *%* of these *K*-mers occur zero times in any given sequence. Not all *K*-mers of this length can be equally important and a dimension reduction must be possible. We found that for *K*>6 a reduction to 2000 dimensions gave the best PLS-performance. Thus, for *K*=7 we reduce the coordinate-space from more than 16000 dimensions to 2000 before computing distances. Still, 2000 dimensions is remarkably large, but of course affected by the fact that we want to classify into a huge number of distinct genera. If the training set includes 1800 different genera, it is perhaps not surprising that we need at least this many dimensions to get a proper resolution to discriminate between them. This huge number of categories, as well as the considerable size variation between them seen in Fig. 1, makes this a rather special classification problem with several methodological challenges worthpursuing.

In [19] flat priors were used in their RDP-classifier. The results presented above also employ this strategy, assuming all genera are equally likely to occur in a new 16S sequence. If genera with many sequences in the training set are truly more widespread, this should be taken into account, and priors reflecting the abundance of each genus in the training set should produce better classifications. On the other hand, if a small training sample is due to an unexplored or newly discovered genus frequency-weighted priors supplies no further information to the data. We tested the RDP-classifier and the Multinomial method with both prior strategies on *Trainingset9*. The results were surprisingly similar regardless of priors. For word length eight the misclassified sequences were practically identical for the two cases, both for full-length and fragmented sequences. With this lack of differences we conclude that, unless very good arguments for the opposite can be provided, flat priors should be used. A flat prior means a single parameter (probability) is used for the entire population instead of (Ockham’s razor) favours the simplersolution.

In the results we observed an error-plateau or barrier below which no *K*-mer based method seemed to reach. Data sets like Trainingset9 and SilvaSet will always contain some proportion of questionable classifications partly since the actual relatedness between various genera is unknown, but also because the 16S gene itself is not a flawless marker. Variability between copies within the same genome as well as recombination events have been reported even for this highly conserved gene [33, 34]. If some sequences have been assigned to an incorrect genus from the beginning, classification errors seems unavoidable. Wang et al. tested their naïve Bayes classifier (RDP-classifier) on two different data sets in their work [19] from 2007. They reported the classification errors at genus level as 8.6 *%* and 7.9 *%* for the Bergey corpus and the NCBI corpus, respectively. The difference from our errors for the same method can be explained by a data set effect, presumably the data sets we have been ‘improved’ by eliminating some obvious mis-assignments since 2007. This emphasizes the importance of training data for classification performance [35].

**Fig. 5:**
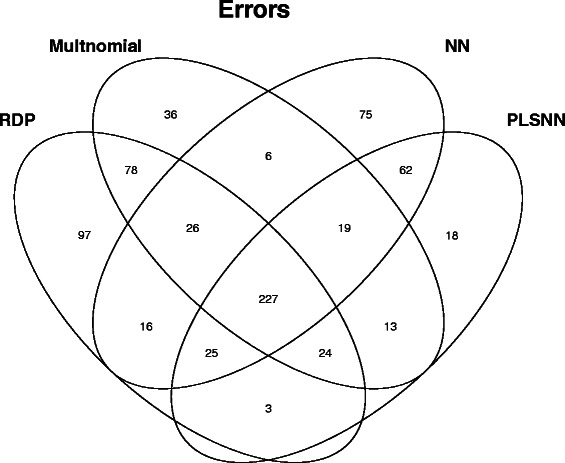
The distribution of errors over methods. The Venn-diagram shows how errors distribute among the different methods. The number in each sector corresponds to the number of mis-classified sequences. The results are from Trainingset9 and full-length sequences

All the sequences that were classified faulty by at least one of the methods were extracted and investigated further. For full-length sequences from Trainingset9 this consisted of 725 sequences, and the errors were distributed over methods as shown in Fig. 5. First, we noticed all methods made some unique errors, from 18 for PLSNN to 97 for RDP. The pairwise relations showed that RDP and Multinomial shared 78 common errors, and NN and PLSNN shared 62. All other pairs had much fewer common errors (3,6,13 and 164), as expected from how the methods are designed. We also noticed that 227 sequences were classified faulty by all methods (center sector), and among these, 176 were assigned to the same genus by all five methods. These 176 sequences belong to 127 unique genera and 42 of these genera contained only two sequences in the fulldata set.

**Fig. 6:**
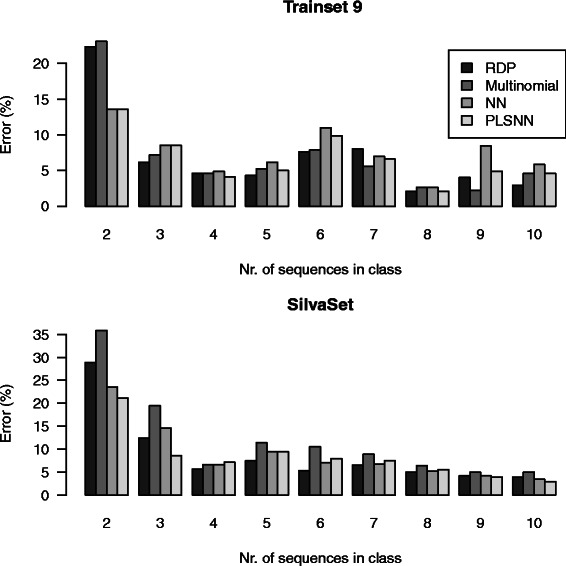
Error distribution over class-sizes. The horizontal axis is the class-size (number of sequences in a genus) and the vertical axis is the error percentage averaged over all genera of the same size. The upper panel is the result from Trainingset9 and the lower panel for the SilvaSet. Only class-sizes up to 10 is shown, for larger classes the error-percentages are small

To investigate further the effect of genus-size, we have in Fig. 6 plotted the error percentages for sizes two to ten. As expected, small genera had elevated risk of being classified wrongly. Genera of size two means there are two sequences in total, one sequence in the training set and one in the test set. Recognizing a genus based on one previously observed sequence is of course very difficult. The genera with only one sequence present (singletons) are not shown as they always will have 100 *%* error. The figure shows that more errors were made for genera consisting of few sequences and this skewness in abundance poses a challenge to all statistical learning methods. One may argue that to improve classifications we need better data more than we need better methods, and that a larger data set is not necessarily a better data set. The SilvaSet is three times larger than Trainingset9 but still relatively more errors were made. We agree that better data is essential, but better data and better methods are also interleaved, since no data set is completely independent of methods, and manual curation is certainly no guarantee against classification errors.

**Fig. 7:**
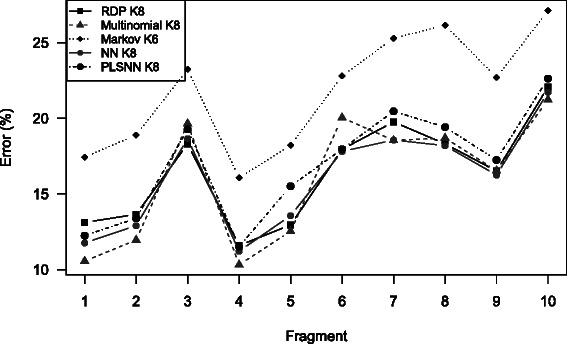
Position specific error. The average error for each method on each of the 10 fragments. The fragments corresponds roughly to (partly overlapping) regions from the start to the end of each 16S sequence. These results are from Trainingset9, but the results from SilvaSet were similar. Hence, the results are discrete values for every *K*-mer length and the connecting lines are merely to aid visual interpretation

In the introduction we mentioned that previous studies show that we should consider all positions along the 16S sequences to get optimal genus-classification. Still, in Fig. 7, we see that some fragments are more informative than others. Fragment four gave a considerably better classification than the other fragments. Please note that we chopped the 16S sequences into ten partly overlapping fragments, all of length 200 bases. Thus, fragment four is located relative to each sequence length, and does not correspond exactly to a hyper-variable region, but is in most cases around region V3-V4, which is known to be the most informative part of the 16S gene. In this perspective it seems likely that there is something to gain from utilizing position-specific information. *K*-mer based methods do not take into account where in the sequence the different words are located, and there may be a potential for improving the methods alongthis line.

## Conclusion

We have compared the popular RDP method to four other *K*-mer based methods with respect to classification of prokaryotes based on 16S sequences. The differences in classification performance are significant, but all methods apart from the Markov method seem to stabilise on a classification error less than 6.6 *%* for word length bigger than seven for full-length sequences. Small extensions to the RDP method, such as counting the frequencies instead of just present/absent, seem to be an advantage, as also pointed out by [22]. On full-length 16S sequences, the Preprocessed nearest-neighbour method stands out as the best, and should be considered for high-precision jobs. With shorter ‘reads’ as input, the naïve Bayes based Multinomial method proves to be the method with least classification errors and therefore the method, out of the five presented methods, which is the optimal option for rapid taxonomicassignments.

The study also reveals the importance of high-quality data for improving the classifications further. All methods seem to level out at some error which is inherent in the various data sets, and it is not likely that improved methods as such will lower this barrier. We have pointed out the special features of this type of data; a large number of categories (genera) in combination with an extreme skewness in their sizes. A key to improve classification is to obtain gold standard training sets in which all efforts have been made to have as few genera as possible with only a few sequences. Increasing the number of representative sequences from one to three or four can greatly increase the classificationaccuracy.

The *K*-mer methods examined here ignore the position specific information that is most likely important to discriminate certain genera. For further improvement of classification, pattern-recognition methods that takes into account position specific information through the 16S sequences may be a good place to start.
